# Epidemiologic Methods to Estimate Insufficient Sleep in the US Population

**DOI:** 10.3390/ijerph17249337

**Published:** 2020-12-14

**Authors:** Girardin Jean-Louis, Arlener D. Turner, Azizi Seixas, Peng Jin, Diana M. Rosenthal, Mengling Liu, George Avirappattu

**Affiliations:** 1 Department of Population Health, New York University Medical Center, 180 Madison Ave, New York, NY 10016, USA; Azizi.Seixas@nyulangone.org (A.S.); Peng.Jin@nyulangone.org (P.J.); Mengling.Liu@nyulangone.org (M.L.); 2 Department of Psychiatry, New York University Medical Center, 145 E 32nd St, New York, NY 10016, USA; Arlener.Turner@nyulangone.org; 3 Population, Policy and Practice Research and Teaching Department, University College London Great Ormond Street Institute of Child Health, 30 Guilford St, London WC1N 1EH, UK; diana.rosenthal@ucl.ac.uk; 4 School of Mathematical Sciences, Kean University, 1000 Morris Ave, Union, NJ 07083, USA; gavirapp@kean.edu

**Keywords:** sleep, sleep health, insufficient sleep, race/ethnicity, relative risk, logistic regression, Poisson regression, population-level estimates

## Abstract

This study explored the divergence in population-level estimates of insufficient sleep (<6 h) by examining the explanatory role of race/ethnicity and contrasting values derived from logistic and Poisson regression modeling techniques. We utilized National Health and Nutrition Examination Survey data to test our hypotheses among 20–85 year-old non-Hispanic Black and non-Hispanic White adults. We estimated the odds ratios using the transformed logistic regression and Poisson regression with robust variance relative risk and 95% confidence intervals (CI) of insufficient sleep. Comparing non-Hispanic White (10176) with non-Hispanic Black (4888) adults (mean age: 50.61 ± 18.03 years, female: 50.8%), we observed that the proportion of insufficient sleepers among non-Hispanic Blacks (19.2–26.1%) was higher than among non-Hispanic Whites (8.9–13.7%) across all age groupings. The converted estimated relative risk ranged from 2.12 (95% CI: 1.59, 2.84) to 2.59 (95% CI: 1.92, 3.50), while the estimated relative risks derived directly from Poisson regression analysis ranged from 1.84 (95% CI: 1.49, 2.26) to 2.12 (95% CI: 1.64, 2.73). All analyses indicated a higher risk of insufficient sleep among non-Hispanic Blacks. However, the estimates derived from logistic regression modeling were considerably higher, suggesting the direct estimates of relative risk ascertained from Poisson regression modeling may be a preferred method for estimating population-level risk of insufficient sleep.

## 1. Background

Insufficient sleep is associated with loss of productivity, increased risk of accidents and injury, and deleterious health conditions such as cardiovascular disease and conditions such as obesity [1]. These associations demonstrate the important health implications of short and long-term loss at the population level. Unfortunately, the wide-spread recommendation for increased sleep time is hampered as studies examining whether the US population has been sleeping progressively less have yielded mixed results [1]. There is ample evidence underlying the notion that discrepant findings may be explained by race/ethnicity, an important proxy for diversity in the sleep experience [2,3,4]. However, given the ongoing debate and the frequency of indiscriminate applications of logistic regression to analyze cross-sectional sleep data, it is also important to explore the accuracy of common analytic techniques while examining the influence of race/ethnicity on self-reported sleep duration.

Discrepancies in estimates of insufficient sleep may be attributed to the common use of logistic regression analysis, which may be suboptimal considering that the prevalence of insufficient sleep in the US population is greater than 10% [2,5]. The extant literature is replete with cross-sectional studies assessing the relationships between exposure and outcome relying solely on logistic regression modeling. Indeed, logistic regression modeling is important in assessing benefits of treatment and risk of exposure, as it yields an odds ratio (OR) while adjusting for potential confounders [6,7]. However, it is not always appreciated that the OR in logistic regression is only an approximation of the relative risk when the disease risk is rare (<10%), and it is generally imprecise when the outcomes are more common [6,8,9]. Several studies addressing diseases occurring in high frequencies have shown that the OR often extensively overestimates the relative risk or prevalence risk [6,10].

The epidemiologic literature has outlined several alternatives for estimating relative risk in cross-sectional analyses of common binary outcomes [6,8,11]. Such alternatives include the use of an adapted Cox regression (also referred to as the Breslow–Cox model), with equal follow-up times assigned to all individuals, and log-binomial regression [6,11], but those methods are not entirely satisfactory. The log-binomial regression model may miss the mark in providing a definite relative risk, while the adapted Cox regression tends to overestimate the variance of the coefficient [6,8]. The most promising method to estimate the relative risk directly seems to be a modified Poisson regression that includes robust variance, albeit more likely to produce very conservative and lower estimates [6,7,8,9,12,13]. Poisson regression is a generalized linear model suitable for data that represent counts of certain events in stochastically independent units, where the number of events is assumed to follow a Poisson distribution [12,13,14]. Since modeling the binomial data using Poisson regression may violate the assumption that the mean and variance are equal, a modified Poisson regression that incorporates the robust error variance procedure was used to optimize its accuracy [7].

The aim of this study was to examine the divergence in population-level estimates of insufficient sleep (<6 h), contrasting values derived from the application of logistic and Poisson regression modeling techniques. Specifically, we hypothesized that the relative risk ratios derived from modified Poisson regression analyses would be lower than those obtained directly from logistic regression modeling, which would yield a more precise estimate of the relative risk of insufficient sleep at the population level. We explored these analytic techniques while examining the potential influence of race/ethnicity, given the evidence that it may explain some of the observed discrepancies [2,3,4].

## 2. Methods

### 2.1. Study Population

This study utilized data from participants in the National Health and Nutrition Examination Survey (NHANES) 2005–2016 [15]. The NHANES is a survey conducted to assess (and track over time) the health and nutritional status of children and adults in the United States using a combination of interviews, physical examinations, laboratory tests, and surveys that include demographic, socioeconomic, dietary, and health-related questions. Findings from NHANES have been used to determine the prevalence of and risk factors for major diseases [15]. Based on research regarding health risks associated with sleep duration [16,17] insufficient sleep was defined as sleep duration less than 6 h, long sleep as sleep duration longer than 8 h [2], and “normal sleep” as sleep duration lasting 7–8 h [18]. Analyses included participants within the age range of 20 and 85 years, excluding those with a missing value for age, gender, education, total household income, body mass index (BMI), systolic blood pressure (SBP), diabetes diagnosis, sleep disorder diagnosis, and sleep duration variables were excluded. Note that we did not exclude cases for missing data on medications known to influence sleep, as data on medication status at that level were not available. The sampling weight of each participant was provided in the NHANES data module instructions [15]. All procedures performed in this study involving human participants were completed in accordance with the ethical standards of the NYU Langone Health Institutional Review Board. 

### 2.2. Statistical Analysis

The continuous variables were expressed as mean with standard deviation (SD) and categorical variables were expressed as percentages (%). The logarithmic transformation of continuous variables was considered if the original variable distribution deviated from the expected Gaussian distribution. Differences in characteristics of participants between Blacks and Whites were compared by the weighted two-sample *t*-test for continuous variables and the weighted chi-squared test for categorical variables, respectively. We compared sociodemographic characteristics (age, sex, education, total household income), medical factors (body mass index, systolic blood pressure, and diabetes), and sleep-related measures (sleep duration, sleep disorder diagnosis) between the racial/ethnic groupings (non-Hispanic White vs. non-Hispanic Black).

Logistic regression was performed to model the association between race/ethnicity and insufficient sleep, adjusting for age, sex, education, income, obesity (BMI), hypertension (SBP), diabetes, and sleep disorders, and the resulting odds ratio (OR) was transformed into an estimated risk ratio (RR). As a comparison, a modified Poisson regression that handles violation of Poisson distribution assumption for common binary outcomes, adjusting for age, sex, education, income, BMI, SBP, diabetes, and sleep disorder, was also performed to model the association between race and insufficient sleep, to estimate the relative risk directly. The modified Poisson regression that incorporates the robust error variance procedure was used to optimize accuracy of the estimates [7]. Specifically, the R-package “sandwich” was implemented to obtain the robust standard errors. We compared the estimated relative risk between Black and White participants on insufficient sleep within specific age groupings: 20–30, 31–40, 41–50, 51–60, 61–70, and >70 years. The 95% confidence interval (CI) was provided for each estimated relative risk. Given 80% power and a fixed sample size, (minimum sample size among the six age groupings), the minimum detectable RR was calculated as 1.62 for logistic regression and 1.50 for modified Poisson regression, at 0.05 significance level. The sampling weights provided by NHANES were applied in all analyses, and *p*-value smaller than 0.05 was considered statistically significant. All statistical analyses performed using software R version 3.6.1 (http://www.r-project.org) [19].

Contrast Between Logistic and Simple Poisson Regression Modeling for a binary outcome

The models:

Logistic regression

(1) $$\log\left(\frac{\pi }{1-\pi }\right)=\alpha_{1}+\beta_{1}x,$$

Poisson regression

(2) $$\log\left(\pi \right)=\alpha_{2}+\beta_{2}x,$$

where π=Pr(Y=1|X=x) and x=0 or 1.

## 3. Results

**Figure 1:**
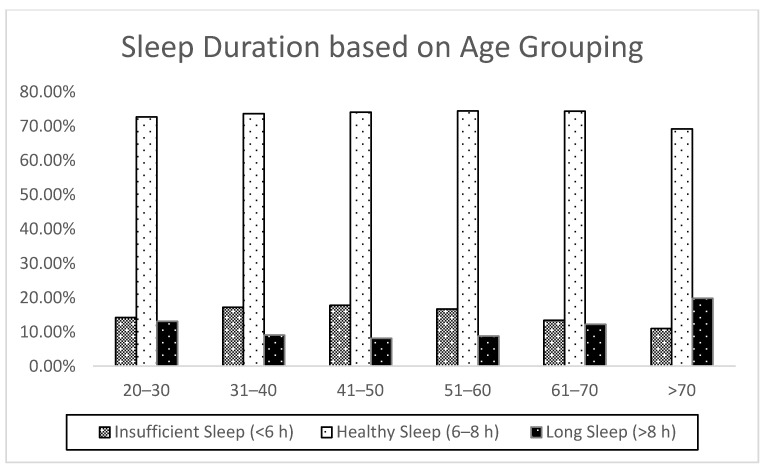
Self-reported sleep duration based on age grouping in the total sample (*n* = 15,604).

**Figure 2:**
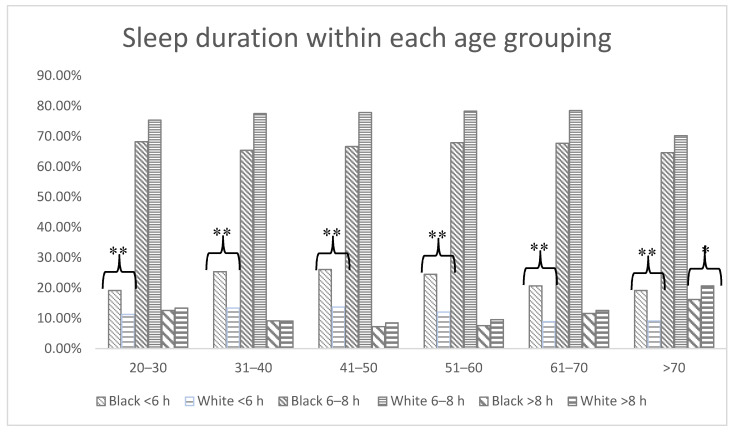
Self-reported sleep duration based on age grouping among non-Hispanic Black (*n* = 4888) and non-Hispanic White (*n* = 10,176) participants. * = *p* < 0.05, ** = *p* < 0.01.

A total of 15,064 participants (4888; 32.4% non-Hispanic Blacks and 10,176, 67.6% non-Hispanic Whites) were included in the analysis. Demographic and health-related characteristics of Blacks and Whites are provided in Table 1. Among all participants, 49.2% were male and 50.8% were female. In Table 2, the proportions of insufficient sleepers, normal sleepers, and long sleepers are provided for all participants, Black participants, and White participants, respectively. In the total sample, the age grouping >70 had the lowest proportion of insufficient sleepers, and the middle-aged grouping (41–50 years) had the highest proportion of insufficient sleepers (Figure 1). The proportion of insufficient sleepers observed among non-Hispanic Black participants was higher than that observed for non-Hispanic White participants across all age groupings (Figure 2).

In Table 3, we provide a summary of the results generated from both the logistic and modified Poisson regression modeling approaches (RRs with 95% CIs). The RRs characterized the association between race/ethnicity and insufficient sleep within each age grouping, adjusting for the effects of age, sex, education, income, BMI, SBP, diabetes, and sleep disorder diagnosis. The estimated RRs were higher for logistic regression compared to Poisson across all age groupings. The age group that demonstrated the most notable difference between RR estimates was the one ranging from 41–50 years (logistic regression estimates RR: 2.59; 95% CI: 1.92, 3.50 vs. Poisson regression estimates RR: 2.06; 95% CI: 1.70, 2.50). The age grouping that demonstrated the least remarkable difference between RR estimates was the one ranging from 20–30 years (logistic regression estimated RR: 2.12; 95% CI: 1.59, 2.84 vs. Poisson regression estimated RR: 1.86; 95% CI: 1.49, 2.32) (Table 3).

## 4. Discussion

Epidemiologic research assessing benefits of treatment and risk of exposure to diseases tends to utilize logistic regression modeling primarily because of its ability to approximate an adjusted relative or prevalence risk with adjustment for confounders [6,7]. However, the extant literature indicates that it may be suboptimal for health conditions or diseases with relatively high prevalence. In those instances, logistic regression modeling overestimates the odds ratios, and is only considered appropriate for diseases with rare occurrences (<10%) [6,7]. It has been suggested the more precise estimate would emanate from the prevalence or relative risk [7,11] (which are mathematically identical [6]) to the odd ratios, and that it is essential to utilize a statistical model that estimates the prevalence/relative risk directly, while preserving the advantages of logistic regression [11]. In that regard, the use of Poisson regression has been a promising alternative. The Poisson distribution in Poisson regression modeling suggests that the events should be ‘rare’, but there is no strict limit on the maximum number of events possible [12,14], making it likely to estimate relative risk directly while preserving the advantages of logistic regression and eliminating the disadvantage of overestimation in occurrences >10%. Unfortunately, there has not been a consensus as to which analytic approach is optimal to assess the prevalence/relative risk. In this study, we tested the precision of estimates of insufficient sleep, in the NHANES dataset, as a real-world example for estimating the prevalence/risk ratios using epidemiologic data.

In this examination of over 15,000 non-Hispanic Blacks and non-Hispanic Whites participating in NHANES, approximately 15% of the sample were insufficient sleepers, while 73% slept 6–8 h. Middle-aged adults (40–51 years) had the lowest proportion of long sleepers while older adults (>70 years) had the highest proportion of insufficient sleepers. The most notable differences in the analytic approaches were observed among middle-aged adults, while the least notable differences were noted among younger adults. This pattern seems to mirror the evidence that a higher proportion of middle-aged adults had insufficient sleep compared with younger adults, pointing to the increased overestimation noted among the latter group. Regarding potential racial/ethnic effects, non-Hispanic Blacks had a higher proportion of insufficient sleepers regardless of age groupings, compared with non-Hispanic Whites. Unsurprisingly, given the high prevalence of insufficient sleep overall, the relative risk ratios derived from both the logistic and Poisson regression analyses demonstrated that non-Hispanic Blacks had a higher risk for insufficient sleep than non-Hispanic Whites across all age groupings. Overall, the results support our hypothesis that the relative risk ratios derived from transforming the odds ratios from the logistic regression were substantially higher than those derived directly from Poisson regression analyses.

Our results comparing the estimated relative risks from logistic and Poisson regression modeling are consistent with previous research [6,7,8,10,12]. For instance, a previous study [10] comparing the modified Poisson regression and logistic regression conversion procedure in a bovine virus, which was present in 24% of the sample, revealed that Poisson regression analysis estimated the relative risk of seropositivity at 2.30 (95% CI: 1.27–4.15), while the use of logistic regression analyses resulted in a higher relative risk of 3.31 (95% CI: 1.55–4.69) [10]. Similarly, our analysis comparing insufficient sleep across six age groupings showing proportions ranging from 19.2–26.1% among non-Hispanic Blacks and 8.9–13.7% among non-Hispanic Whites, resulted in higher estimated relative risks converted from odds ratio derived from logistic regression, compared with estimates derived directly from Poisson regression analysis. More specifically, applying a modified Poisson regression yielded an estimated relative risk of insufficient sleep among non-Hispanic Blacks ranging from 1.84 (95% CI: 1.49, 2.26) to 2.12 (95% CI: 1.64, 2.73), while logistic regression estimated relative risk ranged from 2.12 (95% CI: 1.59, 2.84) to 2.59 (95% CI: 1.92, 3.50).

Our findings indicate that the conversion of odds ratio to relative risk using logistic regression modeling can still lead to an overestimation of the risk estimate that seems to grow as the proportion of cases increases. Additionally, since long sleep is also detrimental to health, our analyses also explored potential differences among long sleepers (>8 h; data not shown). There were no significant estimated RRs from application of the logistic regression modeling. However, the RR for adults > 70 years old from modified Poisson regression (RR: 0.78: 95% CI: 0.62, 0.98) was significant (*p* = 0.03). The finding that the occurrence rates of long sleep fell close to or under the <10% rare level in our sample, lends credence to the notion that the divergence in estimates occurs as the risk of disease increase. These results represent a real-world demonstration of a consistent pattern of over-estimation that aligns well with the extant literature suggesting the application of logistic regression modeling to estimate the prevalence/incidence of diseases with common outcomes increases the difficulty of the relative risk interpretation from the odds ratios [8,20], even with adequate conversion. However, when we used a modified Poisson regression that includes robust variance to estimate the relative risk directly, we observed a greater likelihood of obtaining more conservative estimates [6,7,8,9,12].

## 5. Conclusions

Given that policy decisions, which have important social, political, and economic ramifications, are often made based on the estimates of disease incidence and prevalence, it is essential to ensure that the analytic techniques used to model epidemiologic data are robust and sound. In our approach, we estimated the prevalence of insufficient sleep, an important public health concern [1,2], and the explanatory role of race/ethnicity in understanding health and wellness as a real world example to assess the divergence in population-level estimates contrasting applications of logistic and Poisson regression modeling. In sum, the relative risk ratios derived from both the logistic and Poisson regression analyses demonstrated that non-Hispanic Blacks had a higher risk for insufficient sleep than non-Hispanic Whites across all age groupings, although the logistic regression converted estimates of relative risk were considerably higher. Despite the fact that a tenuous agreement has been reached regarding the use of prevalence/relative risk over odds ratios [7,8,10,11,12], a consensus has not been fully embraced regarding the most robust model to estimate the prevalence/relative risk in common diseases. The results of our study suggest that a direct estimate of the prevalence/relative risk as derived from Poisson regression modeling is a preferred method for estimating the relative risk of insufficient sleep at the population level.

## Figures and Tables

**Table 1:** Demographic and health-related characteristics of participants in the NHANES study.

	Non-Hispanic Black	Non-Hispanic White	*p*-Value ^c^
Participants (*n*)	4888	10,176	
Female Sex	51.82%	50.38%	0.001
Education			0.001
Less than 9th grade	5.05%	4.05%	
9–11th grade	18.04%	12.02%	
High school or equivalent	26.58%	23.96%	
Some college or associate degree	33.14%	31.88%	
College graduate or above	17.18%	28.10%	
Total household income			0.001
$0 to $44,999	58.74%	49.38%	
$45,000 to $99,999	29.93%	29.48%	
$100,000 and over	11.33%	21.14%	
Sleep disorders	25.43%	32.44%	0.001
Diabetes	16.57%	10.56%	0.001
Age (mean, SD)	48.37 (16.97)	51.69 (18.54)	0.001
BMI (mean, SD) ^a^	30.52 (7.84)	28.78 (6.76)	0.001
Underweight	17.40 (0.90)	17.44 (0.91)	0.323
Normal	22.46 (1.71)	22.42 (1.69)	0.819
Overweight	33.32 (6.99)	31.77 (5.98)	0.001
SBP (mean, SD) ^b^	127.34 (19.53)	123.25 (17.83)	0.001
SBP < 120 mm Hg	110.02 (6.91)	109.40 (7.37)	0.005
SBP ≥ 120 mm Hg	138.90 (16.44)	136.03 (14.87)	0.001
Sleep duration (mean, SD)	6.69 (1.65)	7.11 (1.42)	0.001

^a^ Body mass index (BMI: underweight is defined as BMI < 18.5, overweight is defined as BMI ≥ 25, and normal range is in between. ^b^ Systolic blood pressure (SBP) ^c^ *p*-values were calculated based on weighted two-sample *t*-tests or weighted chi-squared tests.

**Table 2:** Self-reported sleep duration between Non-Hispanic Blacks and Non-Hispanic Whites within each age grouping (years).

	Non-Hispanic Black: *n* (%)			Non-Hispanic White: *n* (%)			Total Population: *n* (%)		
	<6 h	6–8 h	>8 h	<6 h	6–8 h	>8 h	<6 h	6–8 h	>8 h
20–30	185 (19.2)	656 (68.2)	121 (12.6)	186 (11.3)	1243 (75.3)	221 (13.4)	371 (14.2)	1899 (72.7)	342 (13.1)
31–40	202 (25.4)	519 (65.4)	73 (9.2)	226 (13.4)	1310 (77.5)	154 (9.1)	428 (17.2)	1829 (73.7)	227 (9.1)
41–50	215 (26.1)	548 (66.6)	60 (7.3)	229 (13.7)	1306 (77.8)	142 (8.5)	444 (17.8)	1854 (74.1)	202 (8.1)
51–60	223 (24.5)	618 (67.9)	69 (7.6)	185 (12.1)	1195 (78.3)	146 (9.6)	408 (16.7)	1813 (74.5)	215 (8.8)
61–70	181 (20.7)	592 (67.7)	101 (11.6)	126 (8.9)	1105 (78.5)	178 (12.6)	307 (13.4)	1697 (74.4)	279 (12.2)
>70	101 (19.2)	339 (64.6)	85 (16.2)	202 (9.1)	1562 (70.2)	460 (20.7)	303 (11.0)	1901 (69.2)	545 (19.8)

**Table 3:** Estimated risk ratios derived from logistic regression and modified Poisson regression modeling.

Age Grouping	N	Logistic Regression		Modified Poisson Regression	
		Estimated Risk Ratio ^1^ (95% CI)	*p*-Value ^2^	Estimated Risk Ratio ^1^ (95% CI)	*p*-Value ^2^
20–30	2612	2.12 (1.59, 2.84)	0.01	1.86 (1.49, 2.32)	0.01
31–40	2484	2.18 (1.65, 2.87)	0.01	1.84 (1.49, 2.26)	0.01
41–50	2500	2.59 (1.92, 3.50)	0.01	2.06 (1.70, 2.50)	0.01
51–60	2436	2.36 (1.71, 3.25)	0.01	1.94 (1.57, 2.41)	0.01
61–70	2283	2.34 (1.57, 3.48)	0.01	1.95 (1.49, 2.56)	0.01
>70	2749	2.45 (1.65, 3.63)	0.01	2.12 (1.64, 2.73)	0.01

^1^ non-Hispanic White is considered as the reference group; ^2^ significance level of 0.05.
